# The Impact of the IL-10 Gene Polymorphism on mRNA Expression and IL-10 Serum Concentration in Polish Lupus Patients

**DOI:** 10.3390/ijms25105511

**Published:** 2024-05-18

**Authors:** Agnieszka Żak-Gołąb, Paweł Cieślik, Urszula Siekiera, Dariusz Kuśmierz, Antoni Hrycek, Michał Holecki

**Affiliations:** 1 Department of Internal, Autoimmune and Metabolic Diseases, School of Medicine, Medical University of Silesia, 40-055 Katowice, Polandgenmar@ka.onet.pl (A.H.);; 2 Regional Blood Donation and Treatment Center, 40-074 Katowice, Poland; 3 Department of Cell Biology, School of Pharmacy, Medical University of Silesia, 41-200 Sosnowiec, Poland

**Keywords:** IL-10 polymorphism, mRNA expression, IL-10, systemic lupus erythematosus

## Abstract

Systemic lupus erythematosus (SLE) is a complex autoimmune disease characterized by the production of autoantibodies against a lot of nuclear components. Despite many studies on the genetic background of this disease, the pathogenesis remains unclear. The aim of the study is to comprehensively evaluate the polymorphism of the IL-10 promoter gene, its mRNA expression, and the serum IL-10 concentration of SLE female patients and females age-matched controls. Analyzing the association between the level of the tested cytokine and the polymorphism genotype-1082; -819; -592, we found statistically higher serum IL-10 levels in SLE patients compared to in healthy controls (11.9 ± 2.2 pg/mL vs. 9.4 ± 1.7 pg/mL, accordingly; *p* < 0.0001). We did not find statistically significant differences in the gene polymorphism of IL-10 among SLE patients and controls. The most significant observation derived from our study is that IL-10 mRNA transcripts are upregulated in SLE patients compared to in healthy controls (*p* < 0.0001). According to our results, the presence of the IL-10 genetic polymorphism has no clinical significance for the development of SLE, and subsequent differences in mRNA and IL-10 concentration results from the influence of other factors which should be the subject of further research.

## 1. Introduction

Systemic lupus erythematosus (SLE) is a complex autoimmune disease characterized by the production of autoantibodies against a lot of nuclear components, that causes damage to multiple organ systems. Despite decades of research, the pathogenesis of SLE remains unexplained [1,2]. The participation of estrogen and environmental factors are postulated, in particular viral infections, ultraviolet radiation, and chemical factors [3,4]. Big data genomic assays have transformed our understanding of SLE. The impact of genetic susceptibility is well supported by the predominance of lupus in families. Gene-wide association studies have found more than one hundred risk loci for SLE [5]. The problem is related to its multi-genic nature and the complex way of inheritance [6].

Cytokines play an important role in the modulation of the immune response and deserve special attention in the formation and development of SLE, among which not only their elevated concentration is important, but also the imbalance between their pro- and anti-inflammatory influence. Cytokines are mediators that play an important role in immune response. The dysregulation of homeostatic cytokine levels has been implicated in the etiology of autoimmune diseases [7,8]. Recently, a lot of interest has been directed towards interleukin 10 (IL-10) in SLE pathogenesis [9,10]. It plays a key role during innate and adaptive immune responses, because it has a wide variety of functions in T, B, natural killer, and dendritic cells, as well as in macrophages. On the one hand, IL-10 exerts anti-inflammatory effects by inhibiting the synthesis of pro-inflammatory cytokines in activated macrophages and T cells, and also has proinflammatory properties and can enhance the proliferation and differentiation of B cells and increase the production of tissue-damaging antibodies [11]. The plasma levels of IL-10 are increased in SLE patients compared to the controls. Elevated IL-10 serum levels have been associated with the increased activity of SLE [12]. 

The IL-10 gene is located on chromosome 1 at 1q31-32. There are many genetic variants of the IL-10 gene; however, the most studied are three single nucleotide polymorphisms (SNPs)-1082(G/A), -819(C/T), and -592(C/A) which form three predominant haplotypes (GCC, ACC, and ATA) [13]. Polymorphisms in the promoter region of the IL-10 gene can affect the expression of the IL-10 cytokine which can lead to changes in immunologic reactions. The dysregulation of IL-10 contributes to autoimmunity [14]. In some studies, the IL-10 gene promoter (rs1800896, rs1800871, and rs1800872) has been shown to alter IL-10 mRNA and protein expression levels [15,16]. Some studies indicate the association between the polymorphism in the promoter region of the IL-10 gene with altered expression of its cytokine and susceptibility for SLE [13,17,18,19]. On the other hand, the ATA haplotype formed by -1082A, -819T, and -592A may play a protective role in SLE in the Chinese population [20]. 

Understanding the etiopathogenesis of SLE may contribute to taking appropriate preventive measures and implementing treatment that minimizes the risk of the development of the disease. Cytokine blockades (TNF-α, IL-6) have been successfully developed to treat rheumatoid arthritis, while barely any anti-cytokine therapies have been introduced into clinical practice as drugs for SLE patients. Therefore, a better understanding of the initiation and progression of SLE may provide suitable novel targets for therapeutic intervention. Many studies assess the impact of the IL-10 polymorphism on SLE development but there are no reports on the simultaneous assessment of the gene polymorphism, mRNA expression, and IL-10 concentration among lupus patients. 

The aim of the study is to discuss the involvement of the polymorphism of the IL-10 promoter gene, its mRNA expression, and the serum IL-10 concentration of SLE patients and controls and in alleles, genotypes, haplotypes, and types of secretions subgroups, as well as to assess the relationships between them.

## 2. Results

Blood samples were collected from 67 consecutive SLE women with no comorbidities, aged 27–79 years (mean 51.7 ± 14.4) and 67 healthy female controls aged 22–81 years (mean 43.1 ± 16.5). We found a statistically higher serum IL-10 concentration in SLE patients compared to healthy female controls (11.9 ± 2.2 pg/mL vs. 9.4 ± 1.7 pg/mL; accordingly, *p* < 0.0001). The differences were also statistically significant when we divided the groups according to genotypes, haplotypes, and secretion level (Table 1).

We investigated three distinct polymorphic regions of IL-10, including -592; -1052; and -819. The distribution of all the genotypes and alleles (co-dominant, dominant, recessive, and over-dominant) in the SLE patients and the healthy controls was in HWE and is illustrated in Table 1. In our study, the presence of the IL-10 genetic polymorphism (positions -1082, -819, and -592) has no clinical significance for the development of SLE (Table 2).

Instead of no statistically significant differences in genotype distributions and allelic frequencies for SLE patients and controls, we found a significant difference in mRNA copies (according to 1 billion 18s rRNA copies) in the SLE patients compared to the healthy controls (*p* < 0.0001) in the whole group, and when dividing participants by genotypes and participants with ACC/ATA, GCC/ACC haplotypes (Table 3).

## 3. Discussion

The pathogenesis of SLE is still not fully understood. SLE involves the interaction between genetic and environmental factors [21]. Genetic factors are a key component in the etiology and pathogenesis of SLE, mainly involving genes with an immune-regulating function. In recent years, many studies have shown that genetic polymorphisms in cytokines genes were associated with SLE. IL-10 is a multifunctional cytokine that has anti-inflammatory properties, demonstrated by its ability to downregulate antigen presentation and macrophage activation. IL-10 production is known to be strongly influenced by genetics [22,23]. However, in spite of the number of genetic studies performed, no definitive result about its involvement in SLE susceptibility was achieved. Some works showed significant associations between IL-10 microsatellites or SNPs with SLE susceptibility or with the development of certain clinical or immunological features, while other studies indicated that this polymorphism did not appear to have any relevance in the disease.

In our study, we did not find that the IL-10 polymorphism gene increased susceptibility to SLE. Similar results were reported by Schotte et al., who suggested that SNPs (-1082A, -592C, and -819) alone were not significantly associated with SLE susceptibility [24]. A meta-analysis by Wang provided evidence of an association between the IL-10 polymorphism and an increased risk of SLE in general and in Asian populations, but no association was found in the Caucasian population [25]. However, the association of the IL-10 promoter allele -1082G with SLE in Asian, but not Caucasian, populations was revealed in a meta-analysis by Swapan at all [26]. Some previous studies have shown that the IL-10 promoter allele -1082G is associated with high levels of IL-10 in SLE [18,27]. The prevalence of high IL-10 producers (-1052G allele or GCC haplotype) has been found to be increased in the Asian population [26] and European patients [28,29], although most of the studies conducted in the Caucasian population did not show significant correlations [30,31].

Polymorphisms in promoter regions can produce changes in the affinity of transcription factors, thus altering the messenger ribonucleic acid (mRNA) expression levels of inflammatory cytokines associated with the risk of autoimmune disease development. In the present study, we measured not only serum IL-10 concentration, but also mRNA expression to assess cytokine protein production by immunocompetent cells. The most significant observation derived from our study is that IL-10 mRNA transcripts are upregulated in SLE patients compared to in healthy controls. Compared to controls, SLE patients showed increased IL-10 mRNA and high IL-10 serum levels [32]. Hedrich et al. found an increased IL-10 mRNA expression in T cells isolated from SLE patients [33]. Moreover, Csiszár et al. reported higher numbers of IL-10 transcripts in unstimulated PBMCs from SLE patients compared to control subjects, identifying B cells and monocytes as the primary cellular sources [34]. It has been suggested that assessing the mRNA expression patterns of autoimmunity-associated genes could be clinically useful for the differentiation of SLE patients from other autoimmunity-affected individuals and for disease monitoring [35]. We did not observe differences between alleles and haplotypes in terms of the mRNA expression and serum concentration of IL-10. Similar results were observed by Vázquez-Villamar, who found that in primary Sjögren’s syndrome, patients showed high serum IL-10 levels and mRNA expression; however, no difference was observed between haplotypes [36]. It was suggested that IL-10 expression is controlled on the transcriptional and post-transcriptional levels. IL-10 can be trans-activated by several factor in monocytes, T and B lymphocytes and antigen presenting cells, and it needs further studies. 

The deregulation of the mRNA expression of IL-10 may have a role in SLE susceptibility and activity. The simultaneous measurement of thousands of mRNAs in individual SLE patients has documented the molecular heterogeneity of SLE. These discrepancies may be due to differences in the cell type analyzed, the study design (including the quantification and interpretation methods of mRNA levels), and the clinical characteristics of the patients included in each study. Some studies postulate a role of epigenetic mechanisms in tissue-specific control of IL-10, which include phosphorylation of histone H3 in the proximal IL10 promoter of murine macrophages, specific chromatin structure, inducible histone modifications, and CpG-DNA methylation patterns in T helper cells that correlate with IL-10 expression [37,38,39,40]. Consequently, these advocate for standardized and cost-effective technical procedures for a more homogeneous evaluation and comparison of gene expression profiles with potential clinical use in SLE patients.

Our results are consistent with previous findings in SLE patients showing increased serum IL-10 levels [41,42].

However, there were some limitations of our study: the small size of the study groups and the inability to compare the results to other Polish investigations. We can only characterize the studied group of SLE women descriptively and we cannot currently provide other demographic characteristics of the studied SLE women. It should also be noted that only a few polymorphisms in the IL-10 gene were considered. 

Differences about the influence of genetic polymorphisms can be attributed to different races, genetic heterogeneity, and environmental influences on the gene. Therefore, further studies are needed to clarify the role of IL-10 gene polymorphism in the pathogenesis of SLE. 

## 4. Materials and Methods

Blood samples were collected from 67 consecutive SLE women aged 27–79 years (mean 51.7 ± 14.4) and 67 healthy female controls aged 22–81 years (mean 43.1 ± 16.5). Blood samples were collected from SLE women and healthy female controls. The patients were diagnosed by rheumatologists and fulfilled the criteria for the classification of SLE according to SLICC (2012) [43]. They were required to have been diagnosed at least 2 months prior. Patients were recruited in accordance with the order of admission to the clinical ward in 2016–2018, which shows the randomness of the group selection. The project applies only to women because at the time of the study only women were under the care of the clinic. The control group was recruited from healthy workers according to the statement. All of them had had SLE for several months, with no or mild activity (SLEDAI 2K: up to 5 score), and without organ involvement—especially the kidneys and the hematopoietic system. They were treated with chronic immunosuppression (most often with prednisone at a dose of up to 20 mg/day, and in some cases additionally with azathioprine). The control group was recruited from age-matched healthy workers. 

This study was approved by the local independent bioethics committee and all patients were required to give their consent. Each participant read information about the study and signed informed consent to participate in it. Patients and the control group had time to ask questions and, if there were any, they received a comprehensive answer. Fasting peripheral blood samples were collected and stored at −80 °C until assay.

In the present study, the IL-10 gene polymorphism at positions -1082 G/A, -819 C/T, and -592 G/A was investigated in SLE patients and compared with the polymorphism in healthy subjects. Additionally, the concentration of IL-10 mRNA converted for 1 million copies of 18srRNA and the concentration of IL-10 were compared. 

DNA was extracted from ethylenediamine tetra-acetate (EDTA) anticoagulated blood using a conventional salting-out procedure. Cytokine gene polymorphism identification was defined for IL-10(1082 G/A, 819 C/T, and 592 C/A). The PCR-sequence-specific primer (PCR-SSP) was performed using a CYTOGEN test. The amplification conditions were the following: hot start at 96 °C, one cycle of 96 °C for 130 s, 63 °C for 60 s, nine cycles of 96 °C for 10 s, 63 °C for 60 s, 20 cycles of 96 °C for 10 s, 59 °C for 50 s, and 72 °C for 30 s. During the electrophoresis in Tris-Borate plus EDTA buffer (150 V/15 min), PCR products (10 µL) were separated on 2.5% agarose gels stained with ethidium bromide. The PCR products were visualized under UV at a wavelength of 302 nm. 

RNA was extracted from the whole blood (collected in test tubes that contained K2 EDTA). The total RNA was isolated from cells by using the Quick-RNA MiniPrep kit (Zymo Research, Irvine, CA, USA) in accordance with the manufacturer’s protocol. Ribonuclease inhibitor was added to the RNA extracts at a concentration of 10 U per 100 mL of RNA solution. Quantitative and qualitative assessments of the extracts were conducted through absorbance measurements at a 260 nm wavelength (assuming that 1.0 OD260 corresponds to 40 μg RNA in 1 mL of extract). Spectrophotometric measurements were performed using an HP8452A spectrophotometer (Hewlett Packard^®^).

To evaluate the number of mRNA copies of the IL-10 in the extracts of total RNA, the RT qPCR method was used. The 18S rRNA gene was used as a constitutive reference gene. Quantitative analysis was carried out by DNA Engine OPTICON (MJ Research, Warsaw, Poland) Fluorescence Detector. The QuantiTect SYBR Green RT-PCR Kit (Qiagen, Germantown, MD, USA) and specific complementary primers to amplify the sequence were used to run the reaction. Primers sequences were designed using Primer Express^TM^ Version 1.0 computer software (ABI Prism, Foster City, CA, USA) based on the data from MEDLINE (https://www.ncbi.nlm.nih.gov; 15.06.2018) (Table 4).

A one-stage RT-qPCR reaction was performed in a 10 μL reaction mixture with 200 ng of total RNA and 0.5 μM of final primer concentration for each forward and reverse primer. Each reaction was carried out in triplicate. In all the reactions, 18S rRNA was applied as an internal control for the RT qPCR. The reverse transcription was performed at 50 °C for 30 min. This stage was followed by incubation at 95 °C for 15 min to inactivate reverse transcriptase (Omniscript and Sensiscript) and active HotStarTaq DNA polymerase. The PCR reaction consisted of 45 cycles of amplification as follows: 15 s denaturation at 94 °C, 30 s annealing at 60 °C, and 10 s extension at 72 °C. The final elongation stage was performed at 72 °C for 10 min. 

The specificity of all amplified samples was confirmed by melting curve analysis. The PCR products were subjected to a gradual temperature increase starting at 50 °C and terminating at 95 °C. The thermal denaturation of the amplicons was represented by rapid fluorescence decrease due to SYBR Green I from double-stranded PCR products. The temperature at which this denaturation occurs is unique to each amplicon and is termed the melting temperature (Tm). The experimentally obtained Tm were compared to the theoretical values.

A commercially available fragment of the β-actin gene (TaqManTemplate Reagent, Applied Biosystems, Warrington, Cheshire, UK) was used as an external standard necessary to determine absolute gene expression. A fragment of the β-actin gene was amplified simultaneously with the samples in five concentrations; i.e., 0.6; 1.2; 3.0; 6.0; and 12.0 ng/μL (1 ng of DNA parallels 333 equivalents of genome). Fluorescence intensities of the serially diluted standards were used to draw a standard curve. Comparing the fluorescence intensities of unknown amounts of the targets with a standard curve allowed the calculation of the initial amount of the targets used in RT-PCR.

Plasma IL-10 levels from 67 SLE patients and 67 healthy controls were measured by enzyme-linked immunosorbent assay (ELISA) according to the manufacturer’s instructions (Human IL-10 ELISA Kit, DIACLONE; Besancon Cedex, France). The optical density of the samples was measured using a microplate reader (µQuant^TM^ Microplate; BioTek U.S., Winooski, VT, USA) at a wavelength of 450 nm.

Statistical analyses were performed with Statistica software version 13 (StatSoft, Inc., Tulsa, OK, USA). The results are expressed as the number and percentage of cases, arithmetic means, and standard deviations. The comparison of studied genotypes with Hardy-Weinberg equilibrium (HWE) was evaluated using the chi-squared analysis. The distribution of IL-10 genotypes, haplotypes, and secretion value in SLE patients and in healthy controls was evaluated using the chi-squared Pearson test. The risk of SLE development was shown as the odds ratio (OR) and the 95% confidence interval (95% CI). The IL-10 mRNA and enzyme concentration in SLE and control genotypes, haplotypes, and secretion value groups were compared using the Mann–Whitney test. The level of significance was set at *p* < 0.05.

## 5. Conclusions

The influence of the IL-10 gene on the risk of SLE is still not clear. According to our results, the presence of the IL-10 genetic polymorphism has no clinical significance for the development of SLE, and subsequent differences in mRNA and IL-10 concentration suggest that transcription factors (activated by different cytokines or stimuli) and epigenetic factors are involved in the regulation of the IL-10 gene expression. 

A better understanding of these biological factors may provide essential clues to the pathogenic pathways and open new possibilities for more effective therapeutics. IL-10 levels and mRNA transcripts, upregulated in SLE patients, highlight them as potential targets for immunotherapy. Our results suggest the complex mechanism of genetic etiology and indicate possible therapeutic targets in the treatment of SLE.

AŻG conceived the concept of the study. AŻG, MH, and AH contributed to the design of the research. AŻG was involved in data collection. PC, US, and MK analyzed the data. All authors edited and approved the final version of the manuscript.

## Figures and Tables

**Table 1:** IL-10 concentration [pg/mL] in SLE patients and healthy controls. Healthy controls contain alleles, genotypes, haplotypes, and types of secretions.

	N	SLE x ± SD	N	Control x ± SD	*p*
Total	67	11.92 ± 2.19	67	9.44 ± 1.69	<0.0001
1082A/G					
Co-dominant model					
AA	24	11.83 ± 1.61	18	9.67 ± 2.14	<0.0001
AG	29	11.69 ± 2.04	40	9.44 ± 1.66	<0.0001
GG	14	12.52 ± 3.22	9	9.02 ± 0.47	<0.0001
Dominant model					
AG+GG	43	11.96 ± 2.48	49	9.36 ± 1.51	<0.0001
Recessive model					
AA+AG	53	11.76 ± 1.84	58	9.51 ± 1.80	<0.0001
Over-dominant model					
AA+GG	38	12.09 ± 2.32	27	9.45 ± 1.78	<0.0001
819T/C					
Co-dominant model					
TT	1	10.70	1	8.30	-
TC	24	11.80 ± 2.35	23	9.75 ± 2.30	<0.0001
CC	42	12.01 ± 2.14	43	9.30 ± 1.28	<0.0001
Dominant model					
TC+CC	66	11.93 ± 2.21	66	9.46 ± 1.70	<0.0001
Recessive model					
TC+TT	25	11.76 ± 2.31	24	9.69 ± 2.27	<0.0001
Over-dominant model					
TT+CC	43	11.98 ± 2.13	44	9.28 ± 1.27	<0.0001
592A/C					
Co-dominant model					
AA	1	10.70	1	8.30	-
AC	24	11.80 ± 2.35	23	9.75 ± 2.30	<0.0001
CC	42	12.01 ± 2.14	43	9.30 ± 1.28	<0.0001
Dominant model					
AC+CC	66	11.94 ± 2.21	66	9.46 ± 1.70	<0.0001
Recessive model					
AC+AA	25	11.76 ± 2.31	24	9.69 ± 2.27	<0.0001
Over-dominant model					
AA+CC	43	11.98 ± 2.13	44	9.28 ± 1.27	<0.0001
Haplotype					
ACC/ATA	14	11.76 ± 1.95	10	9.76 ± 2.75	0.0031
GCC/GCC	14	12.52 ± 3.22	9	9.02 ± 0.47	<0.0001
GCC/ATA	10	11.86 ± 2.94	13	9.75 ± 2.01	0.0032
GCC/ACC	20	11.71 ± 1.49	27	9.29 ± 1.48	<0.0001
ACC/ACC	8	11.86 ± 0.89	7	9.73 ± 1.17	0.0059
ATA/ATA	1	10.70	1	8.30	-
Secretion					
Low	23	11.75 ± 1.60	18	9.67 ± 2.14	<0.0001
Intermediate	30	11.76 ± 2.03	40	9.44 ± 1.66	<0.0001
High	14	12.52 ± 3.22	9	9.02 ± 0.47	<0.0001

*p* = test Mann Whitney.

**Table 2:** The IL-10 polymorphism among SLE patients and healthy controls contains alleles, genotype, haplotypes, and types of secretions.

	SLE n (%)	Control n (%)	OR	95% CI	p*
1082A/G					
A	77 (57%)	78 (57%)			
G	57 (43%)	58 (43%)	0.970	[0.598–1.574]	0.9018
Co-dominant model					
AA	24 (36%)	18 (27%)			
GA	29 (43%)	40 (60%)	0.544	[0.250–1.181]	0.1574
GG	14 (21%)	9 (13%)	1.167	[0.414–3.290]	
Dominant model					
AA	24 (36%)	18 (27%)			
GA+GG	43 (64%)	49 (73%)	0.658	[0.315–1.374]	0.2639
Recessive model					
AA+GA	53 (79%)	58 (87%)			
GG	14 (21%)	9 (13%)	1.702	[0.681–4.257]	0.2520
Over-dominant model					
AA+GG	38 (57%)	27 (40%)			
GA	29 (43%)	40 (60%)	0.515	[0.259–1.024]	0.0573
X2 HWE (p)	0.88 (0.348)	3.13 (0.078)			
819T/C					
C	108 (81%)	109 (81%)			
T	26 (19%)	25 (19%)	1.050	[0.570–1.932]	0.8763
Co-dominant model					
CC	42 (63%)	43 (64%)			
CT	24 (36%)	23 (34%)	1.068	[0.524–2.179]	0.9836
TT	1 (1%)	1 (1%)	1.024	[0.062–16.921]	
Dominant model					
CC	42 (63%)	43 (64%)			
CT+TT	25 (37%)	24 (36%)	1.067	[0.528–2.154]	0.8577
Recessive model					
CC+CT	66 (99%)	66 (99%)	.		
TT	1 (1%)	1 (1%)	1.000	[0.061–16.325]	1.0000
Over-dominant model					
CC+TT	43 (64%)	44 (66%)			
CT	24 (36%)	23 (34%)	1.068	[0.525–2.171]	0.8564
X2 HWE (p)	1.41 (0.234)	1.15 (0.284)			
592A/C					
C	108 (81%)	109 (81%)			
A	26 (19%)	25 (19%)	1.050	[0.570–1.932]	0.8763
Co-dominant model					
CC	42 (63%)	43 (64%)			
CA	24 (36%)	23 (34%)	1.068	[0.524–2.179]	0.9836
AA	1 (1%)	1 (1%)	1.024	[0.062–16.921]	
Dominant model					
CC	42 (63%)	43 (65%)			
CA+AA	25 (37%)	24 (35%)	1.067	[0.528–2.155]	0.8577
Recessive model					
CC+CA	66 (99%)	66 (99%)			
AA	1 (1%)	1 (1%)	1.000	[0.061–16.325]	1.0000
Over-dominant model					
CC+AA	43 (64%)	44 (66%)			
CA	24 (36%)	23 (33%)	1.068	[0.525–2.171]	0.8564
X2 HWE (p)	1.41 (0.234)	1.15 (0.284)			
Haplotype					
ACC/ATA	14 (21%)	10 (15%)	0.664	[0.272–1.623]	0.3675
GCC/GCC	14 (21%)	9 (13%)	0.860	[0.293–2.524]	0.7841
GCC/ATA	10 (15%)	13 (19%)	1.372	[0.555–3.390]	0.4919
GCC/ACC	20 (30%)	27 (40%)	1.586	[0.776–3.245]	0.2051
ACC/ACC	8 (12%)	7 (11%)	0.860	[0.293–2.524]	0.7841
ATA/ATA	1 (2%)	1 (2%)	1.000	[0.061–16.326]	under 5 cases
Secretion					
Low	23 (34%)	18 (27%)	0.703	[0.336–1.472]	0.3486
Intermediate	30 (45%)	40 (60%)	1.827	[0.920–3.627]	0.0837
High	14 (21%)	9 (13%)	0.587	[0.235–1.469]	0.2520

Description: p* = Chi2 Person; *p* = Chi-square test for deviation from the Hardy—Weinberg equilibrium (X2 HWE): The frequency of genotypes in the study group was compared with the expected frequency resulting from the Hardy—Weinberg equilibrium using a statistical calculator.

**Table 3:** IL-10 RNA copies converted to 1 billion 18S rRNA copies. Comparison between SLE and control in subgroups of compatible alleles, Haplotypes, and type of secretion; *p* = Mann—Whitney test.

	N	SLE x ± SD	N	Control x ± SD	*p*
Total	67	1333.7 ± 1567.2	67	644.5 ± 434.7	<0.0001
1082A/G					
Co-dominant model					
AA	24	875.42 ± 925.47	18	487.72 ± 249.74	0.0141
AG	29	1355.55 ± 1221.31	40	682.53 ± 400.75	<0.0001
GG	14	2074.00 ± 2600.39	9	788.89 ± 746.50	0.0956
Dominant model					
AG+GG	43	1589.47 ± 1789.82	49	702.06 ± 474.44	<0.0001
Recessive model					
AA+AG	53	1138.13 ± 1113.65	58	622.07 ± 369.80	<0.0001
Over-dominant model					
AA+GG	38	1317.00 ± 724.30	27	588.11 ± 482.88	0.0021
819T/C					
Co-dominant model					
TT	1	393.00	1	268.00	-
TC	24	845.71 ± 892.74	23	695.22 ± 480.06	0.3922
CC	42	1634.93 ± 1803.49	43	626.09 ± 413.58	<0.0001
Dominant model					
TC+CC	66	1347.94 ± 1574.80	66	650.18 ± 435.46	<0.0001
Recessive model					
TC+TT	25	827.60 ± 878.62	24	677.42 ± 477.54	0.3453
Over-dominant model					
TT+CC	43	1606.05 ± 1791.93	44	617.95 ± 412.29	<0.0001
592A/C					
Co-dominant model					
AA	1	393.00	1	268.00	-
AC	24	845.71 ± 892.74	23	695.22 ± 480.06	0.3922
CC	42	1634.93 ± 1803.49	43	626.09 ± 413.58	<0.0001
Dominant model					
AC+CC	66	1347.94 ± 1574.80	66	650.18 ± 435.46	<0.0001
Recessive model					
AC+AA	25	827.60 ± 878.62	24	677.42 ± 477.54	0.3453
Over-dominant model					
AA+CC	43	1606.05 ± 1791.93	44	617.95 ± 412.29	<0.0001
Haplotype					
ACC/ATA	14	888.21 ± 1158.83	10	423.80 ± 137.95	0.0417
GCC/GCC	14	2074.00 ± 2600.39	9	788.89 ± 746.50	0.0956
GCC/ATA	10	786.20 ± 300.46	13	904.00 ± 547.38	0.6482
GCC/ACC	20	1606.90 ± 1393.43	27	575.89 ± 257.45	<0.0001
ACC/ACC	8	936.63 ± 526.75	7	610.43 ± 340.21	0.3357
ATA/ATA	1	393.00	1	268.00	-
Secretion					
Low	23	883.52 ± 945.40	18	487.72 ± 249.74	0.0175
Intermediate	30	1333.33 ± 1206.22	40	682.53 ± 400.75	<0.0001
High	14	2074.00 ± 2600.39	9	788.89 ± 746.50	0.0956

**Table 4:** Nucleotide sequences of primers used in the RT-PCR re action (primers supplied by Oligo IBB PAN).

Transcript Detected	Forward Primer (5′-3′)	Reverse Primer (5′-3′)	Accession Number
IL-10 mRNA	GGAGGAGGTGATGCCCCAAGCTGA	TGCTCCACGGCCTTGCTCTTGT	NM000572
18S rRNA	CAGTTATGGTTCCTTTGGTCGCTC	GTTGATAGGGCAGACGTTCGAATG	M10098

## Data Availability

The data presented in this study are available on request from the corresponding author.
